# *Brain and Behavior*: we want you to share your data

**DOI:** 10.1002/brb3.192

**Published:** 2013-11-28

**Authors:** Maryann E Martone

## Abstract

We at Brain and Behavior are happy, for one, that data sharing is now here.

Like the crazed woman in gothic novels, scientific data have long been relegated to the dark basements and attics of scientific laboratories. However, perhaps these days are over: data, especially big data, are all the rage, along with increasing calls to make the data on which scholarly claims are made into first-class citizens of scholarship. These calls are welcome to some; reviled by others. Many reasons are given as to why we cannot, do not, or should not make data available (e.g., Strasser 2013; Wallis et al. 2013), but I think that the main reason we do not routinely share data is that, until recently, we could not. And because we could not, a system of scholarly communication grew where data were disposable. Literally. Eventually, the boxes piled upon boxes and file cabinets overflowed. With no system in place to find, access, share, and use data, their ultimate fate was usually the basement or, ultimately, the garbage bin. And because scholarly communication drives the entire reward system of academia, from promotion to funding, we created a system where the primary products of research upon which science rest: the data themselves were second-class citizens.

So perhaps we should stop and ask ourselves: If, in some alternative reality, we somehow arrived at the 21st century without any tradition of scholarly communication, what would we invent now that would serve science best? Would it be a system that treated the hard won and often expensive products of our instruments and intellect as disposable by-products? Would we design a system in which researchers were rewarded for keeping their data secret and inscrutable and where many of the products of research funding were never recovered, because no one was rewarded for making them available? Would it be a system that insisted only positive results be reported and encouraged selective use of data to tell a good story (Mueck 2013)?

Or would we perhaps instead design a system where the data were viewed as primary products of research and were an integral part of any communication about them? Or perhaps a system where we recognized that some researchers are excellent at producing data and others at analyzing them and so allowed a marketplace or ecosystem to develop that did not diminish one at the expense of the other? Perhaps we might even insist that data are *the* primary product of research, which serve to anchor an ecosystem of discussion and analysis subsequent to their dissemination, and so require their release before we publish any analysis of them (Birney et al. 2009).

So perhaps because we never could share data on a large scale before the digital revolution, we somehow grew to think it is not necessary or even desirable. No one can possibly understand scientific data except those that produce them, we say. The data are too messy and incomplete to use for anything (although not, apparently, to make claims about them in a paper). If someone wants my data, they can e-mail me (Wallis et al. 2013). Those in favor of data sharing and open data are challenged to defend their stance by showing that it is useful. However, at this point, I think that it is equally incumbent on those who object to show that it is not or cannot be. We have seen the abuses and the biases in our current system (Begley and Ellis 2012; Mueck 2013); perhaps we ought to be open to at least a trial period where we make an effort to determine whether routine publishing data is an exercise in futility or whether it opens a gateway to faster and more impressive discoveries. And that can only be done by making large amounts of data available. Without a significant amount of data, how will we be able to develop the computational and human expertise to deal with the messy, heterogeneous nature of scientific data? How will we know how data might be used to increase transparency and efficiency? We have to start somewhere.

So we at *Brain and Behavior* are happy, for one, that data sharing is now here. Funding agencies around the world are developing policies regarding the availability of research data. For example, the Office of Science and Technology Policy of the US President has declared that agencies will work to develop policies to make the results of federally funded research freely available to the public and for requiring researchers to better account for and manage the digital data resulting from federally funded scientific research (OSTP 2013). Governmental agencies and academic institutions around the world have already invested considerably in the infrastructure required to host research data; literally thousands of databases are available for researchers to deposit their data (Cachat et al. 2012). By and large these resources have been underutilized. With the OSTP mandate and new initiatives like BD2K in the US and the European Human Brain Project, the time has come to kick the tires on these investments and spur the scientific community to both populate and mine these resources. After we have had a few years of data sharing, we can then assess what, when, how, where, and even if the data should be available. If our current way is best, we can always go back to it.

We certainly understand that much work remains to be done to make data a first-class citizen in scholarly communication, including norms and best practices for data citation and tracking. Fortunately, the community has not been idle. Various groups have been working toward developing the appropriate standards for ensuring that data sets are citable as research objects (CODATA-ICSTI Task Group on Data Citation Standards and Practices 2013) and providing metadata standards for doing so (DataCite 2013). Over 25 different groups have convened through FORCE11: the Future of Research Communications and e-Scholarship to produce a consensus draft of data citation principles (http://www.force11.org/node/4381). Thompson Reuters has launched their Data Citation index, to complement their article citation index. The data landscape will likely be volatile for a few more years, with false starts and dead ends before we determine what works and what does not.

We are pleased to announce that we will actively encourage all who publish in *Brain and Behavior* to make their data available, and are planning some incentives to ensure that authors are rewarded for doing so. For example, *Brain and Behavior* will now allow researchers to publish data papers. Data papers will allow researchers to publish a paper describing a data set that will be deposited within a certified data repository. A certified repository is one that is committed to the long-term preservation of data, employs metadata standards and can issue an appropriate identifier, for example, a DOI, to a data set.

What is the difference between a data paper and a regular research paper? A data paper focuses on the data themselves and not their analysis. Data papers will be judged on the perceived value of the data, for example sufficient number of subjects, data quality, and descriptive metadata, and whether the data themselves are in an actionable form. By “actionable,” we mean that they are in a form suitable for machine-based access and not just human consumption. The peer review of these data will therefore likely include both a biomedical researcher and someone who is familiar with data structures. These requirements will mean that researchers will have to spend some time cleaning and annotating their data. Whereas earlier, there was little incentive for researchers to put in this extra effort, with the data paper, the researcher will get a publication and we can use current metrics of tracking citations to measure the impact of the data set. As with our regular paper submissions, *Brain and Behavior* will accept all types of relevant data sets that meet these requirements.

What will be the impacts of widespread sharing of data and full population of data resources? Analysis of public data sets is already resulting in publications (Service 2013) and certain data sharing initiatives are viewed as highly successful, for example, ADNI. But I suspect it will likely be several years before we start to see the tangible fruits of routine data sharing in terms of new types of analyses or insights that make their way into the scientific corpus or are realized into new products or treatments. However, I believe that the intangibles are already here; those of us who run data repositories know that people are looking at data and downloading them. Who knows how many people were inspired to do experiments or were stopped from doing additional experiments because of accessible data? This type of impact is difficult to measure, but is very real. At a minimum, sharing data will increase the transparency of science and diversify the palate from which we can draw inspiration; at the maximum, data sharing will help usher in our brave new world of 21st century scholarly communications and propel scientists to do their job faster and better.
